# Correction to “The new low‐toxic histone deacetylase inhibitor S‐(2) induces apoptosis in various acute myeloid leukaemia cells”

**DOI:** 10.1111/jcmm.70001

**Published:** 2024-11-08

Cellai C, Balliu M, Laurenzana A, et al. The new low‐toxic histone deacetylase inhibitor S‐(2) induces apoptosis in various acute myeloid leukaemia cells. *J Cell Mol Med*. 2012;16:1758‐1765. doi:10.1111/j.1582-4934.2011.01464.x.

In C. Cellai et al, the published article contains errors in Figure 6A. The corrected images for Spleen (control and after treatment with S‐2, magnification 100×) are provided below. The authors confirm that the results and conclusions of this article remain unchanged.

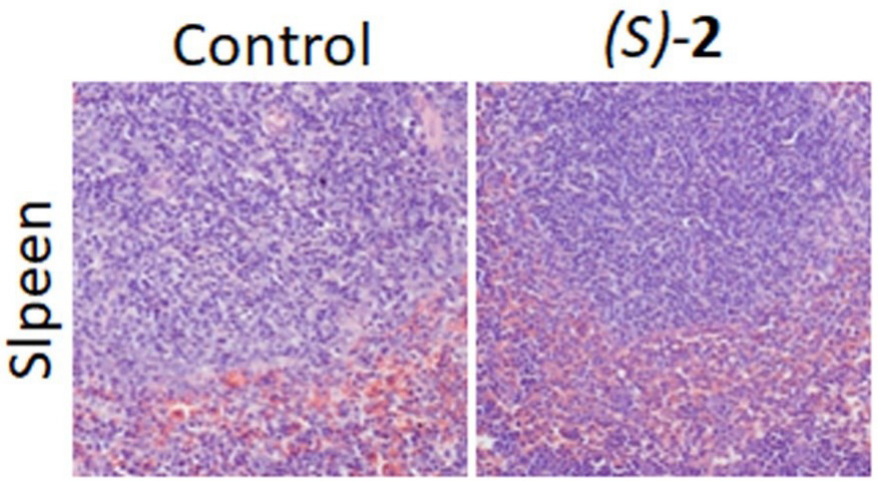

We apologize for this error.

